# Supplementary material for: Combinatorial CRISPR screen identifies fitness effects of gene paralogues
Source: Nat Commun. 2021 Feb 26;12:1302. doi: 10.1038/s41467-021-21478-9 (PMC7910459; doi:10.1038/s41467-021-21478-9)
Supplement: Supplementary file 1 — Supplementary Figures [file 41467_2021_21478_MOESM1_ESM.pdf]

Thompson *et al.*,

Combinatorial CRISPR screen identifies fitness effects of gene paralogues

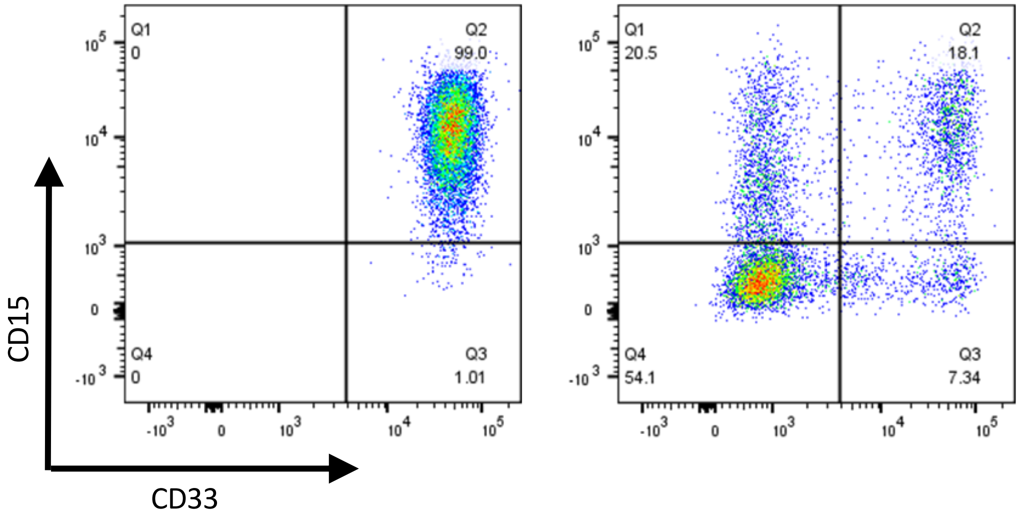

Supplementary Figure 1: Efficient co-disruption of CD15/CD33 using paired gRNA targeting. Molm-13-Cas9 cells were infected with either a non-targeting control virus (left) or a virus containing gRNAs directed against CD15/CD33 (right). At 2 weeks cells were stained with conjugated antibodies against CD15 (APC) and CD33 (PE) and quantified for expression of these markers. Cells were gated on FSC and SCC as shown in Supplementary Figure 2.

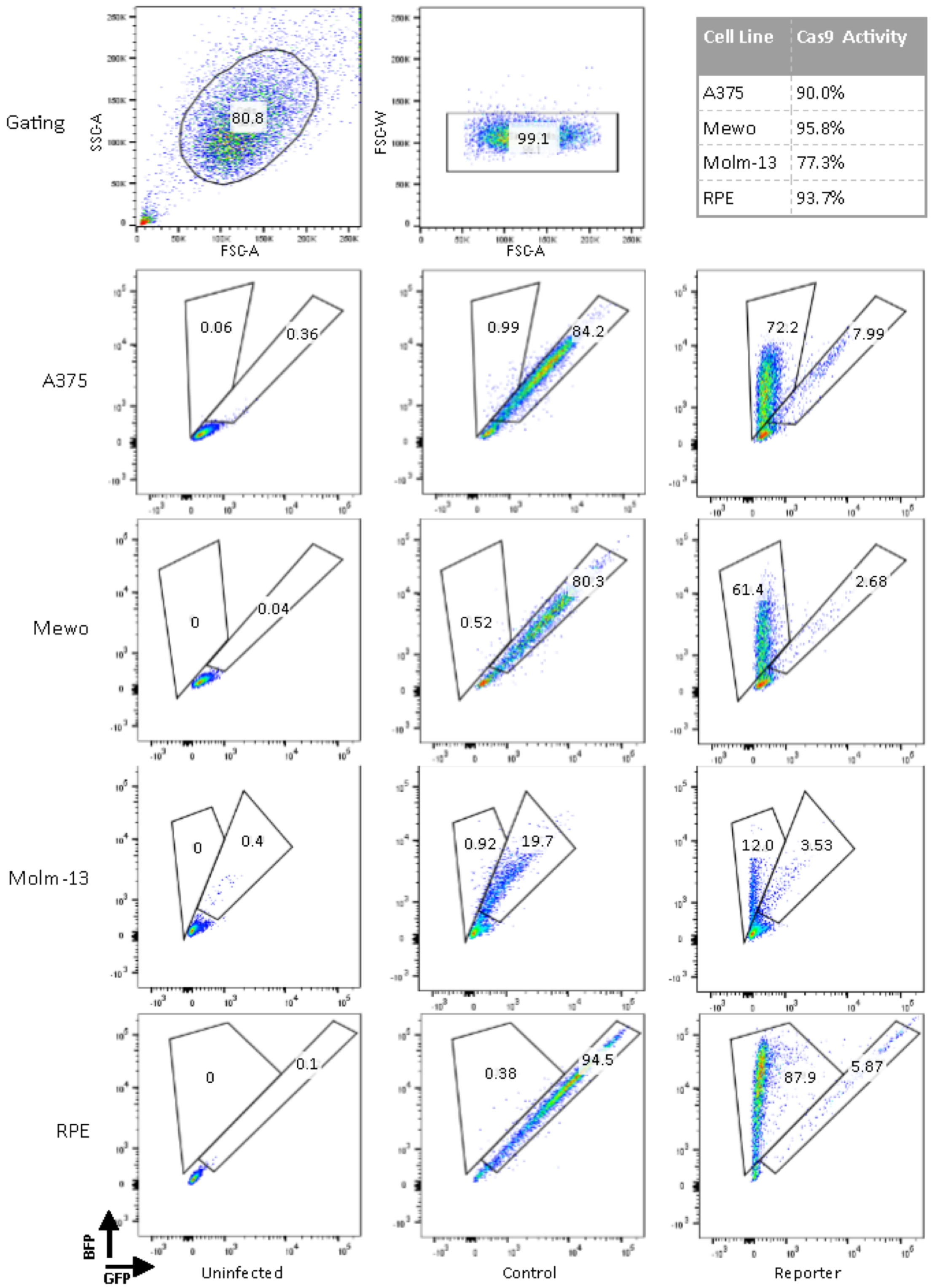

Cell Line	Cas9 Activity
A375	90.0%
Mewo	95.8%
Molm-13	77.3%
RPE	93.7%

Supplementary Figure 2: Cas9 efficiency test of cell lines. To assess Cas9 activity cells were transduced with either a control BFP/GFP expressing virus (central column) or a reporter virus containing BFP/GFP and a guide against GFP (right column). Uninfected cells are shown in the left column. Fluorescence was assessed by FACS 72 hours post transduction. Gating is shown in the top row. Cells expressing Cas9 cleave GFP when infected with the reporter virus, creating a BFP+/GFP- population. The fraction of Cas9 expressing cells was calculated by dividing the % of BFP+ cells by the total % of cells infected. The Cas9 activity for each cell line assayed is shown in the table (top right).

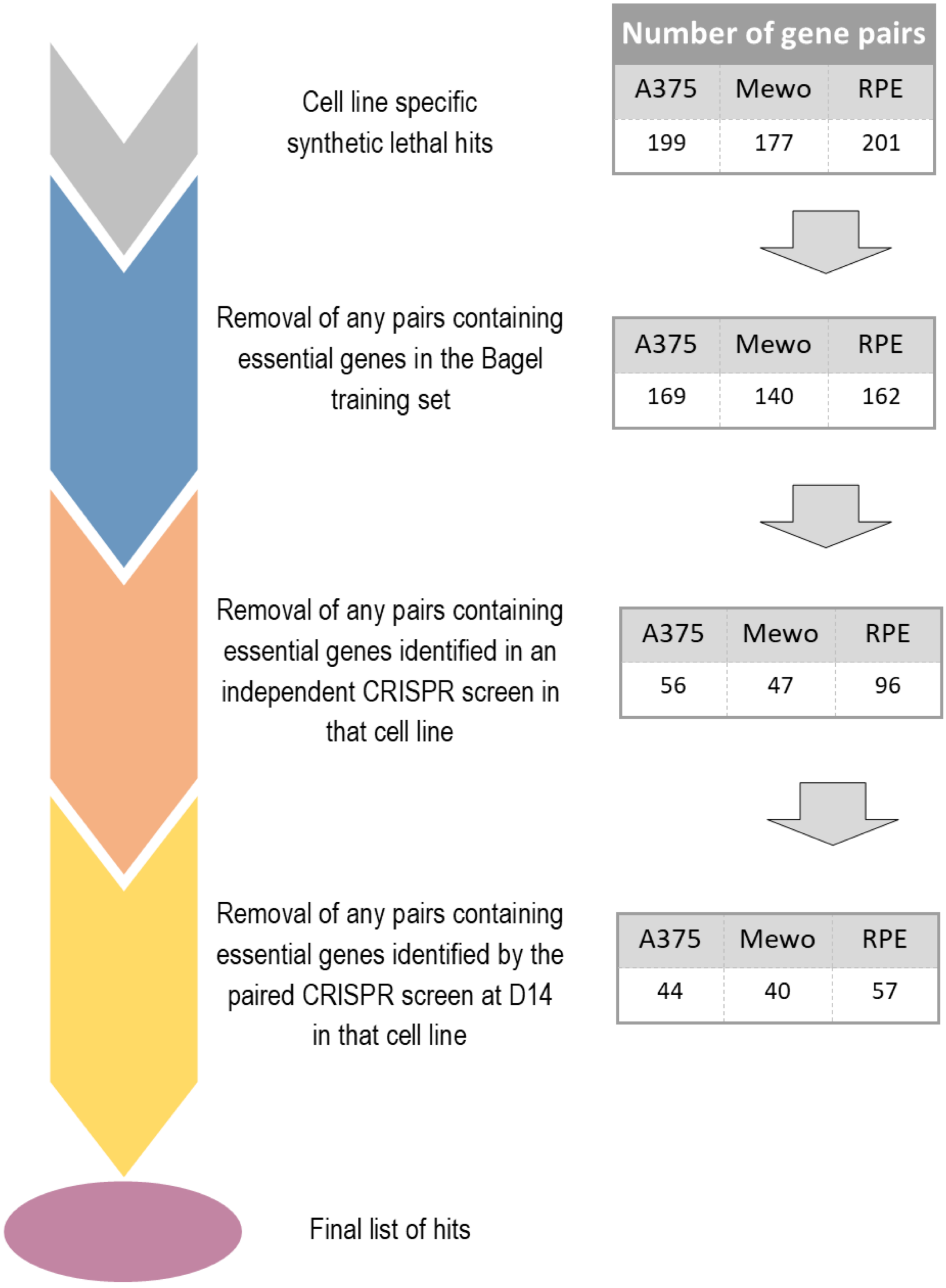

Supplementary Figure 3: Post-screen data filtering and processing to generate the final hit list. All gene-pairs identified as having a potential synthetic lethal interaction in each screen by both the RRA and t-test were selected. From this list, all gene-pairs containing an essential gene were removed (as defined by BAGEL. See **Methods**). Following this, all gene-pairs containing an essential gene identified by either BAGEL or MAGeCK in an independent CRISPR screen in that cell line were removed. Finally, all gene pairs containing an essential gene identified by BAGEL or MAGeCK in the paired CRISPR screen at day 14 were removed (See **Methods**). The numbers on the right indicate the number of genes present at each filtration step in each cell line.

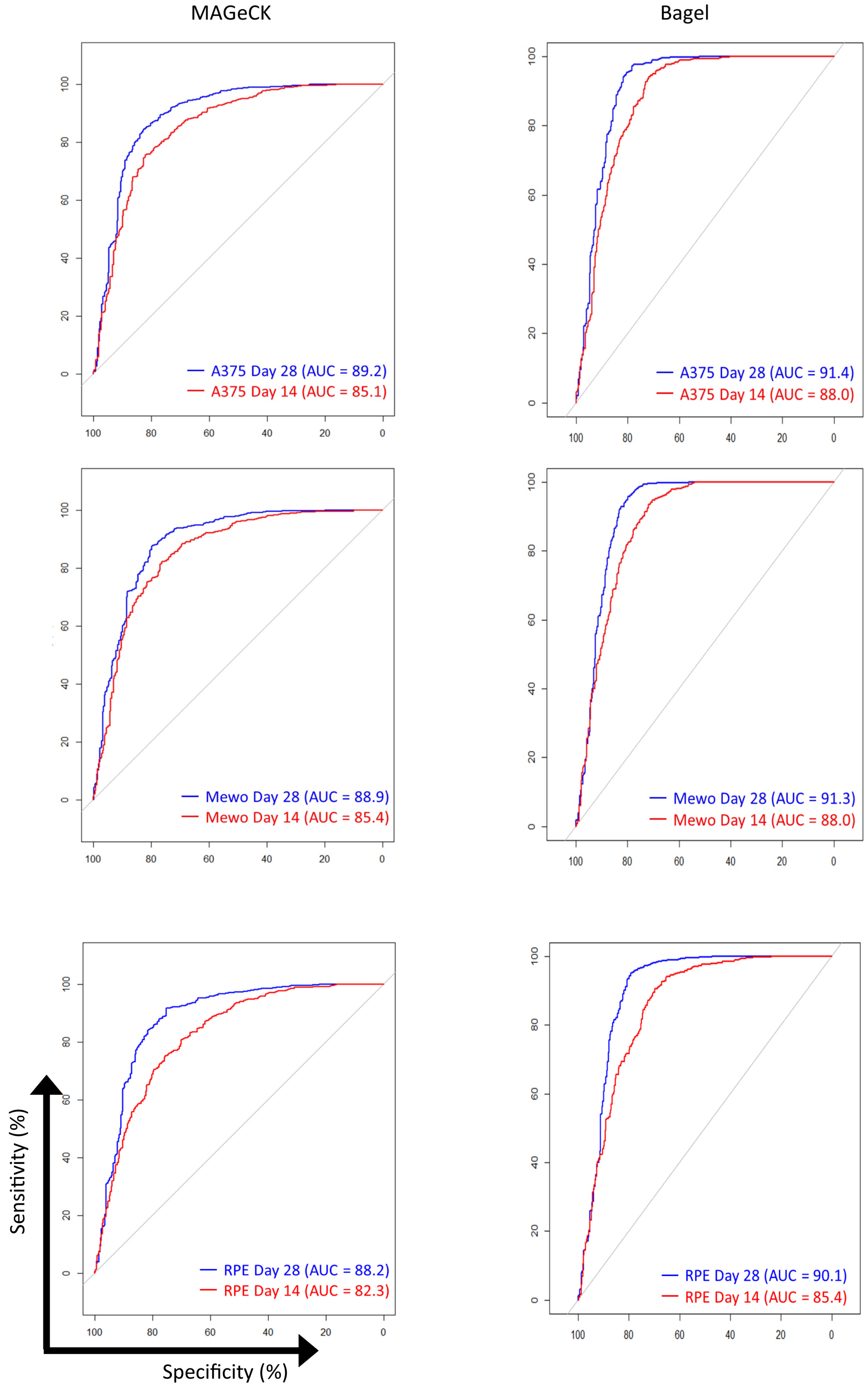

Supplementary Figure 4: Receiver Operator Curves. Data shown for all screens at both the day 14 (red) and day 28 (blue) time point. Plots were generated using the pROC package in R Studio. The predictor for MAGeCK was the gene *p* value; the predictor for BAGEL was the gene Bayes Factor. The outcome in both cases was the gene classification (essential or nonessential). Any gene not belonging to either of these two classes was ignored. The BAGEL essential/non-essential genes were from: http://bagel-for-knockout-screens.sourceforge.net (training essential/non-essential). Hart *el al*., G3 (Bethesda). 2017 Aug 7;7(8):2719-2727. doi: 10.1534/g3.117.041277. Hart *et al*., BMC Bioinformatics. 2016 Apr 16;17:164. doi: 10.1186/s12859-016-1015-8.

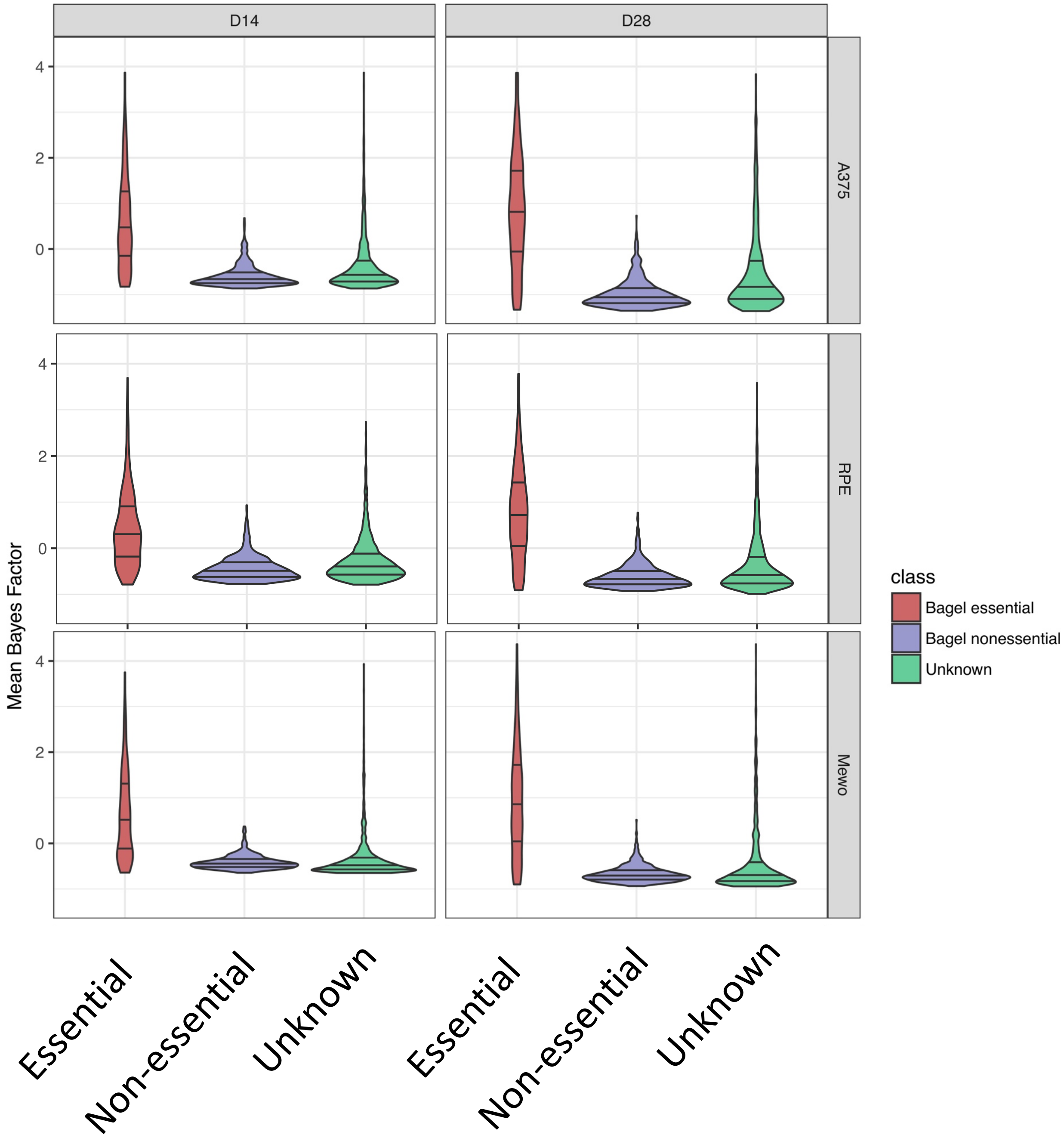

Supplementary Figure 5: Bayes factor distribution of essential and non-essential genes. Mean Bayes factors generated by BAGEL for all single gRNA constructs are shown for each screen at both time-points [Day 14 (D14) and Day 28 (D28)]. All single gene constructs not belonging to the BAGEL training set of essential/non-essential genes were classed as "Unknown". The higher the Bayes factor, the more likely the guide is to be essential; this is reflected by the higher Bayes factors seen in the essential gene class. The horizontal lines indicate the 25th, 50th and 75th quantile. The BAGEL essential/non-essential genes were from: http://bagel-for-knockout-screens.sourceforge.net (training essential/non-essential). Hart *el al.*, G3 (Bethesda). 2017 Aug 7;7(8):2719-2727. doi: 10.1534/g3.117.041277. Hart *et al.*, BMC Bioinformatics. 2016 Apr 16;17:164. doi: 10.1186/s12859-016-1015-8.

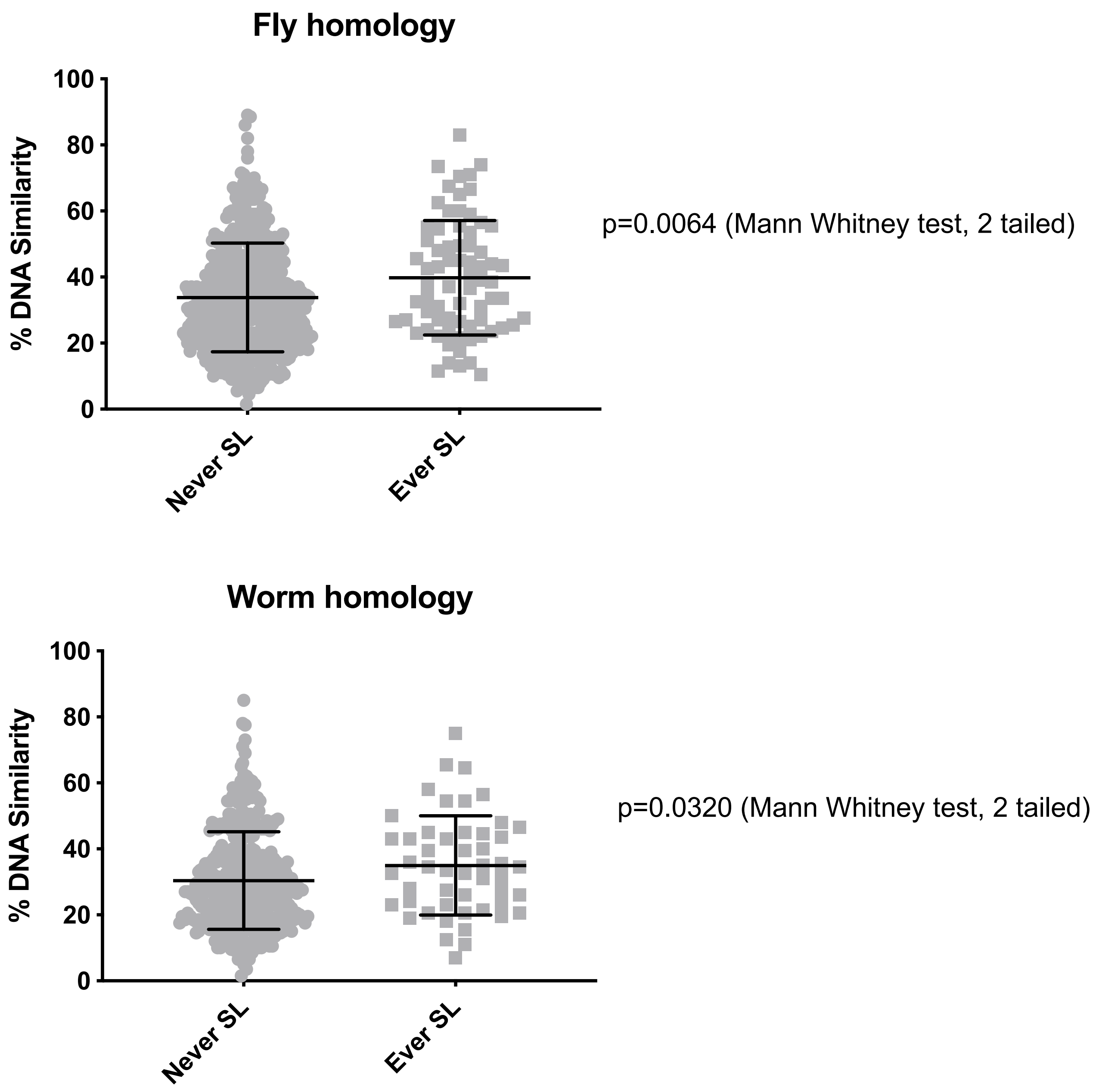

Supplementary Figure 6: The association between sequence conservation and synthetic lethality for paralogous gene pairs. Never synthetic lethal refers to paralogue pairs that are never lethal in any of the cell lines tested while ever synthetic lethal refers to pairs which are lethal in one or more cell lines. Bars represent the mean and standard deviation of each group.

A

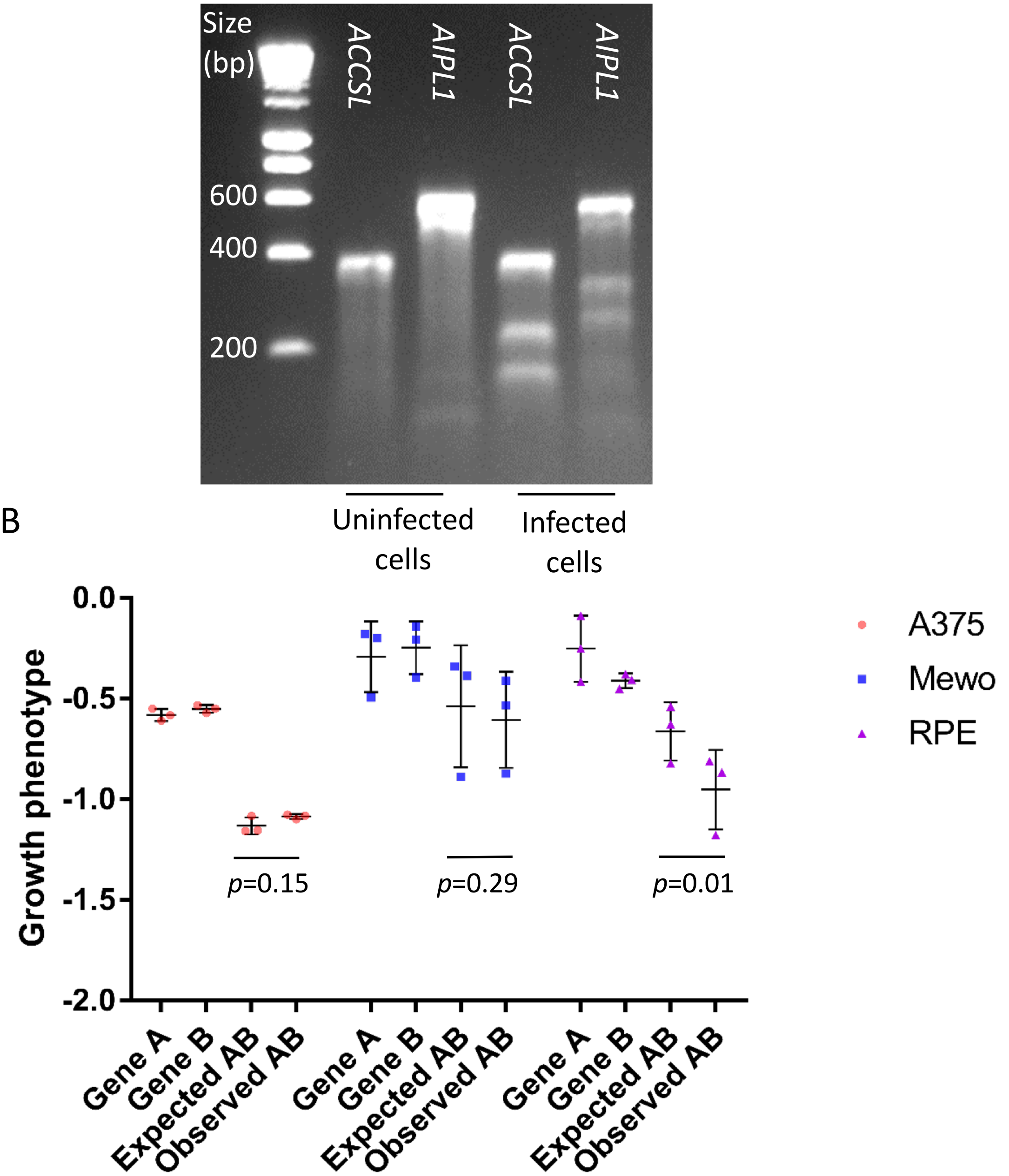

Supplementary Figure 7: Establishing a baseline for a non-interacting gene pair (A) The A375 cell line was transduced with viruses containing guides against *ACCSL* and *AIPL1*. After one week, DNA was extracted and PCR was performed across the expected cut site. The PCR products were then treated with surveyor nuclease. Presence of cleaved PCR fragments indicate gRNA-directed genomic editing. (Expected sizes: *ACCSL* uncleaved-376bp; *ACCSL* cleaved-145/231bp; *AIPL1* uncleaved-572bp; *AIPL1* cleaved-323/249bp). The *ACCSL* and *AIPL1* genes come from a previously released dataset (https://sourceforge.net/projects/bagel-for-knockout-screens/files/). This experiment was performed once. (B) Cells were transduced with two viruses containing *ACCSL*_ BFP and a *AIPL1*_mCherry to give four populations. Population abundance was measured at day 4 and 14; all infected populations were normalised to the uninfected fraction to give a relative abundance. The growth phenotype was calculated by taking the $\log_2$ (fold change (relative abundance)) of the single and double-infected cells at day 14 vs day 4. Gene A is *ACCSL*; Gene B is *AIPL1*; the expected phenotype is the sum of the growth phenotype of Gene A and B; the observed phenotype AB is that of the double positive population. The experiment was performed in technical triplicate; shown is the mean, error bars represent standard deviations; *p* values were generated using a 2-sided paired t-test in Graphpad Prism. Cells were gated on FSC and SCC as shown in Supplementary Figure 2.

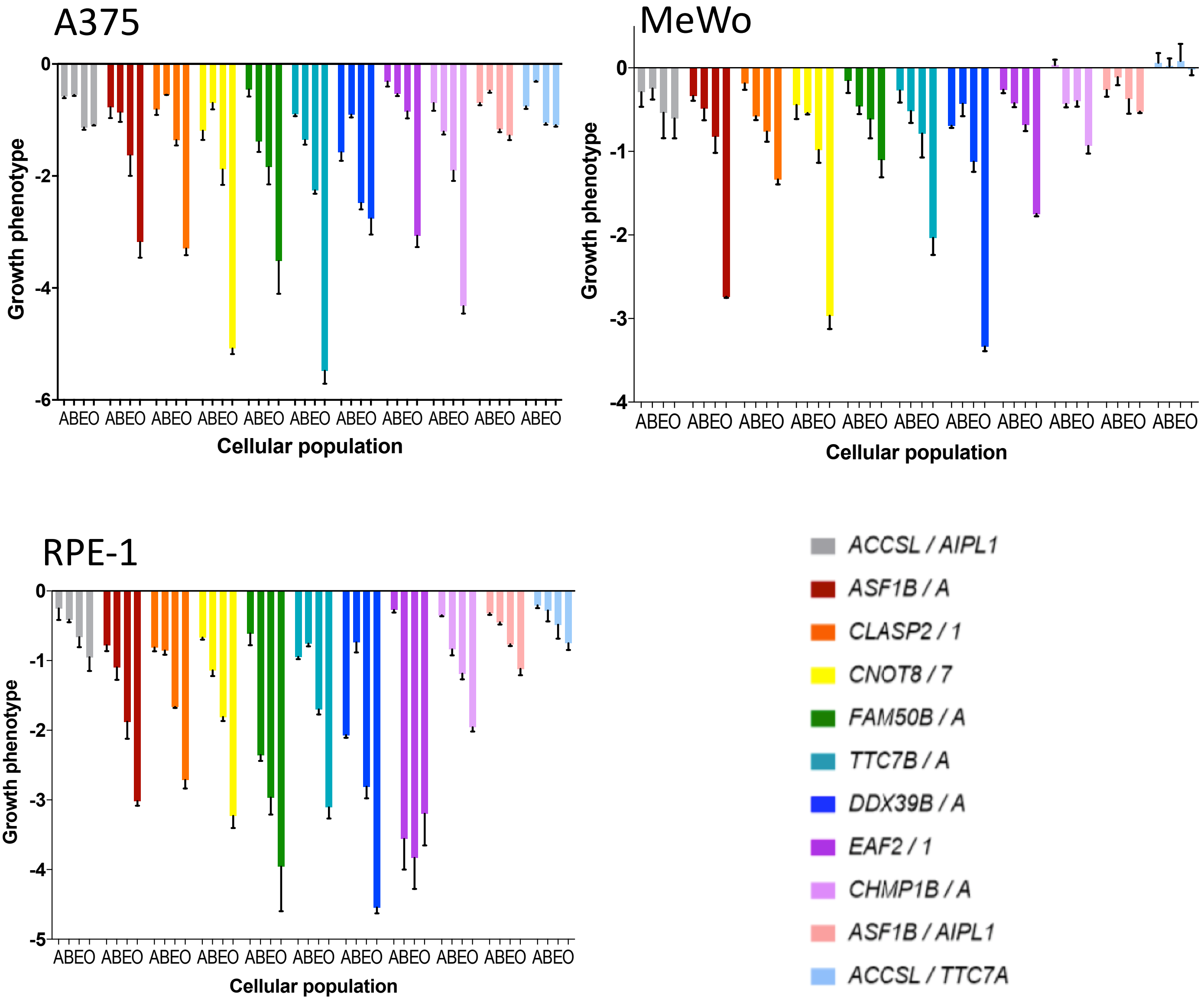

Supplementary Figure 8: Validation of predicted synthetic lethal interactions. Cells were transduced with a gRNA-A_mCherry virus and gRNA-B_BFP virus to give four populations. Population abundance was measured at day 4 and 14. The growth phenotype was calculated by normalising all three infected populations to the uninfected population (relative abundance), then calculating the $\log_2$ of the relative abundance fold change between day 14 and day 4 (A = growth phenotype of gene A; B = phenotype of gene B; E = expected phenotype of the double positive population (sum of gene A and B); O = observed phenotype of the double positive population). Where the observed phenotype is more negative than the expected phenotype, the gene pair is more lethally than expected. These data are the result of three independent transductions. Graphs show the mean, error bars show the standard deviation. Cells were gated on FSC and SCC as shown in Supplementary Figure 2.

Overlap of gene pairs with De Kegel *et al.*,

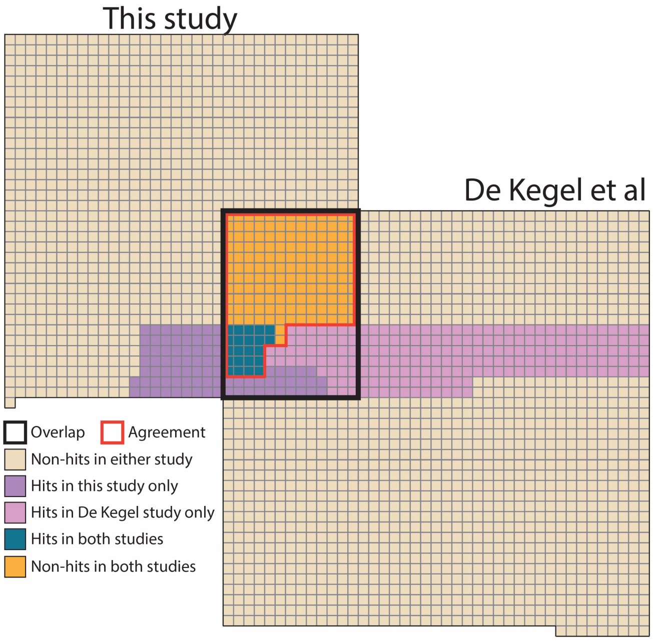

Overlap of gene pairs with Gonatopoulos-Pournatzis *et al.*,

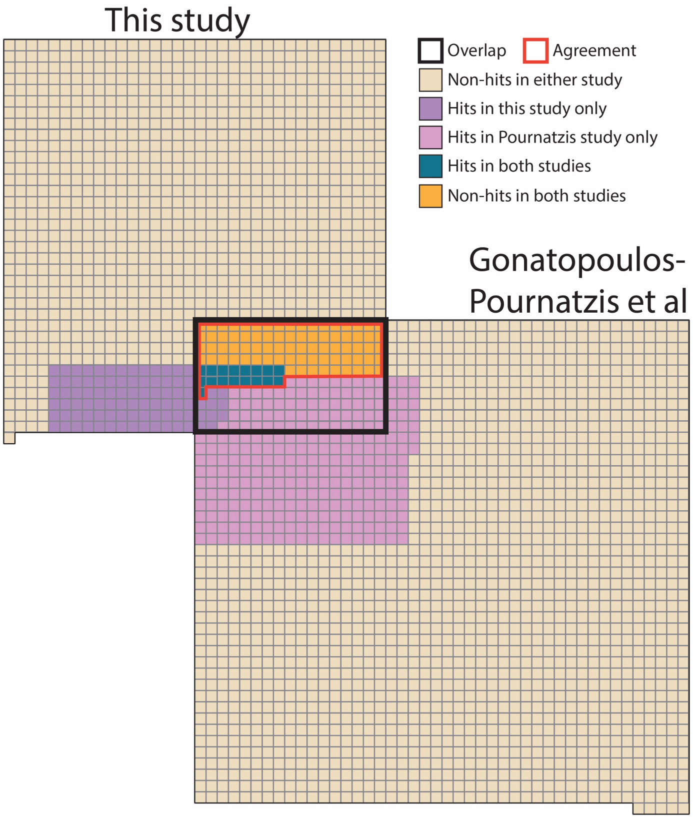

Supplementary Figure 9: Overlap of hits with other synthetic lethal gene sets. We compared the overlap between our 105 (**Supplementary Table 4**) hits with those of two other studies; De Kegel *et al*., (PLoS Genet. 2019 Oct 25;15(10):e1008466) and Gonatopoulos Pournatzis *et al*., (Nat Biotechnol. 2020 May;38(5):638-648). We considered a gene pair a "hit" if it was scored as such in any of the cell lines screened by either our screen or the screens performed by Gonatopoulos Pournatzis *et al*.,. In the same way we used all gene pairs computed to be candidate synthetic lethal interactions by De Kegel *et al*.,.

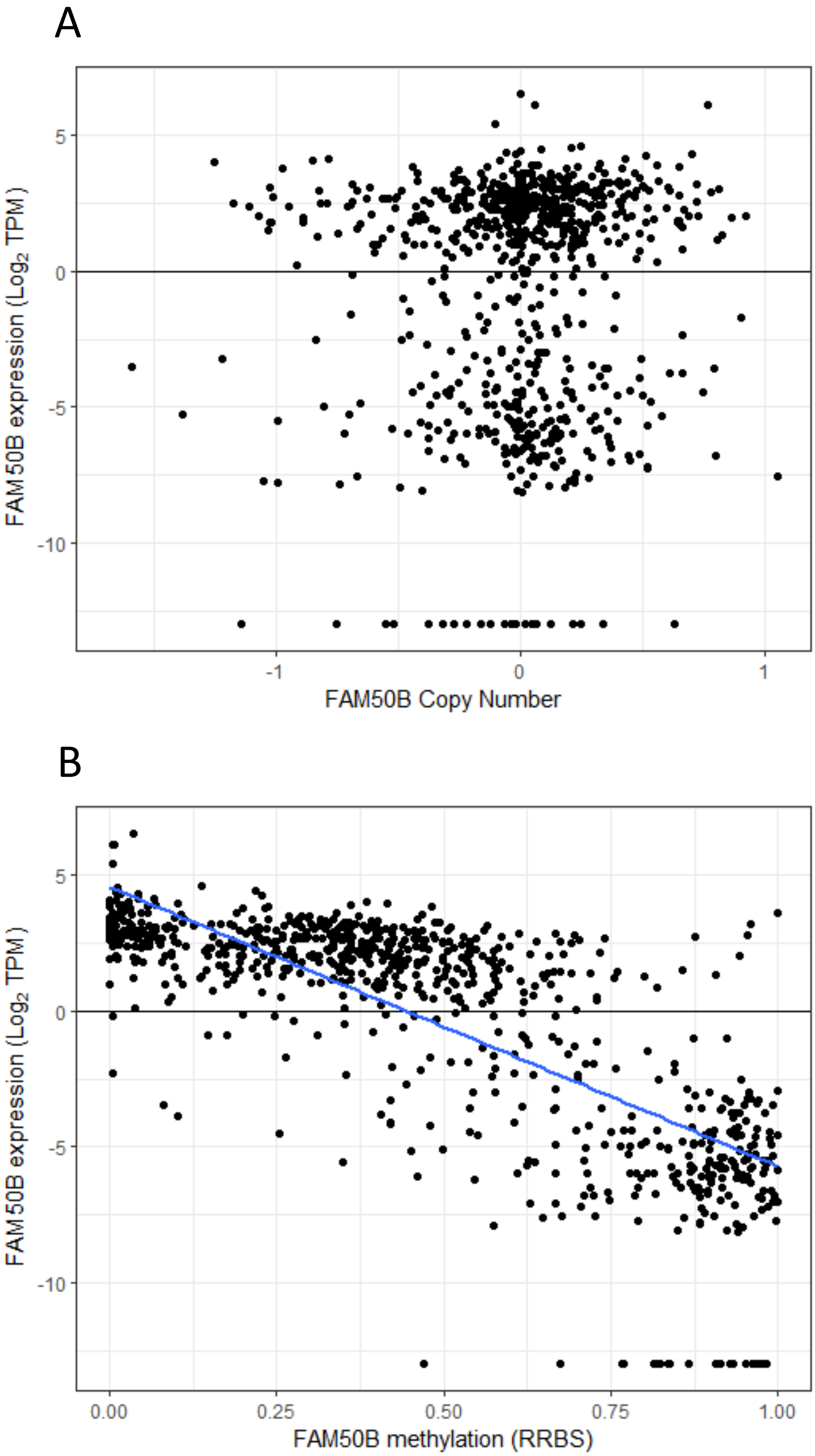

Supplementary Figure 10: Relationship between *FAM50B* expression, methylation and copy number across 831 cancer cell lines. (A) relationship between *FAM50B* copy number (measured using Affymetrix SNP 6.0 arrays) and gene expression. (B) Relationship between methylation fraction (measured using RRBS) and *FAM50B* expression showing a negative correlation between expression and methylation (Pearson's correlation coefficient -0.77, blue line shows linear regression). Data obtained from CCLE (https://portals.broadinstitute.org/ccle).

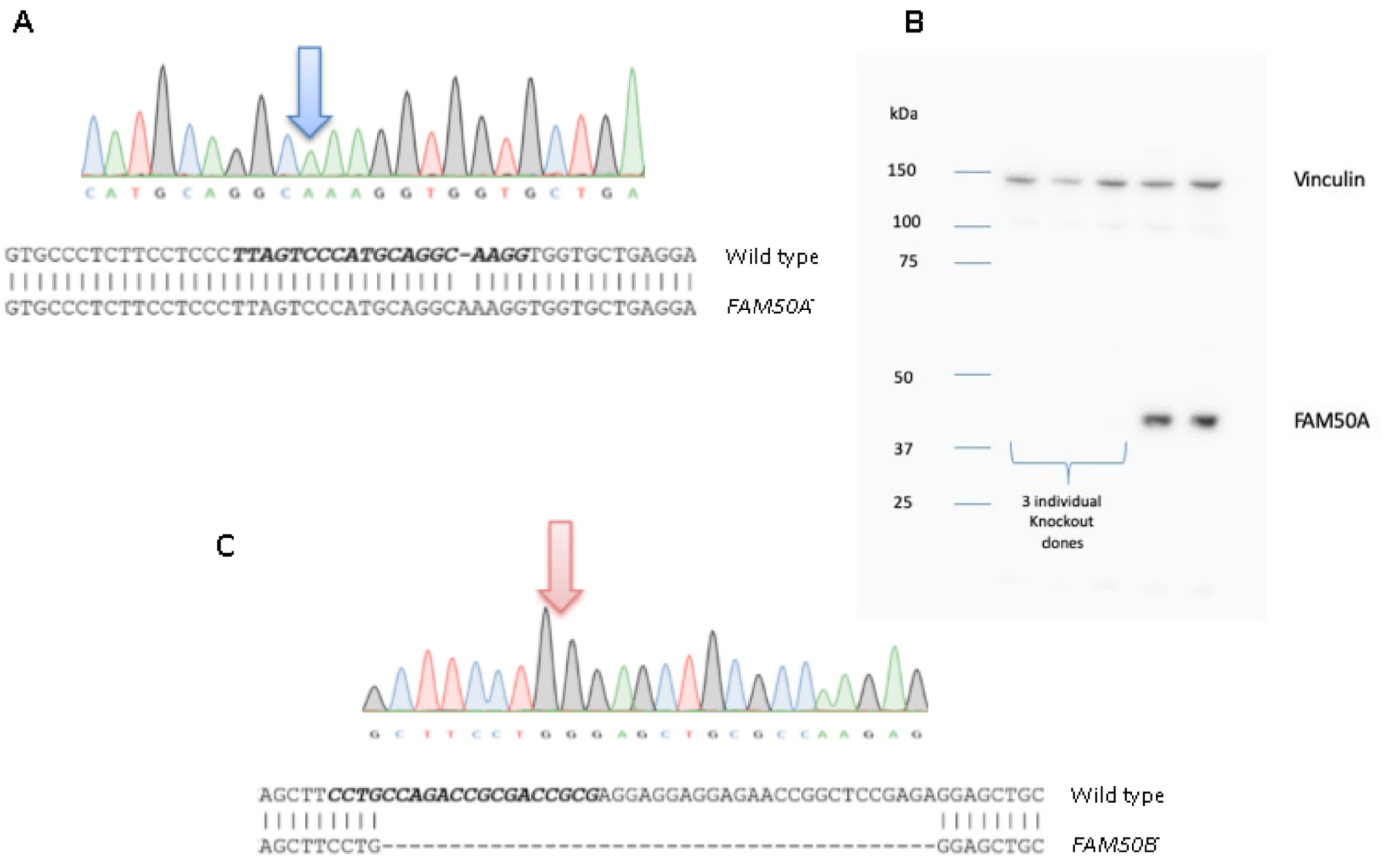

Supplementary Figure 11: Generation and analysis of isogenic *FAM50A* and *FAM50B* knockout cell lines in A375 cells. (A) Sanger sequencing of the *FAM50A* knockout clone used for subsequent experiments, showing a single base pair insertion. (B) Western blot confirming knockout of *FAM50A* in three clones. Two *FAM50B* knockout clones are shown as controls. This Western blot was performed once. The cell lines were further validated using transcriptome sequencing and frameshift editing events were confirmed in both *FAM50A* and *FAM50B* mRNA (see **Methods**). (C) Sanger sequencing of the *FAM50B* knockout clone used for subsequent experiments, showing a 41bp deletion. For A and B the sequence of the gRNA used is shown in bold/italics in each of the sequences.

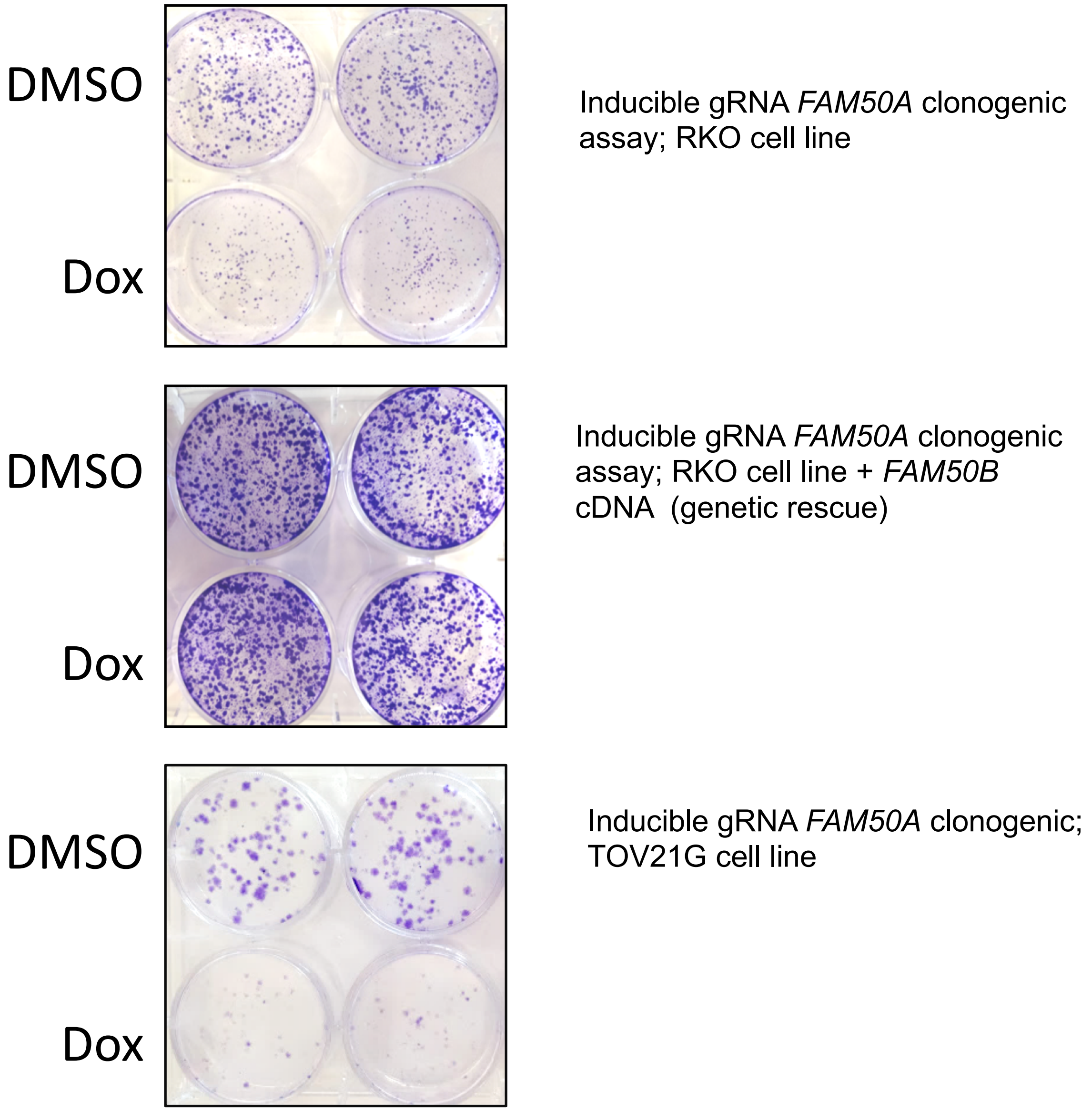

Supplementary Figure 12: Further validation of the synthetic lethal interaction between *FAM50A* and *FAM50B* in cells carrying an inducible *FAM50A* gRNA construct. Cells were seeded at 1000 cells per well. At 24 hours media was changed to either contain Dox (0.1ug/ml) or DMSO. At day 9 (RKO) or day 14 (TOV21G) cells were fixed and stained with crystal violet (see **Methods**). Each of these experiments were replicated at least three times on separate clonal lines.

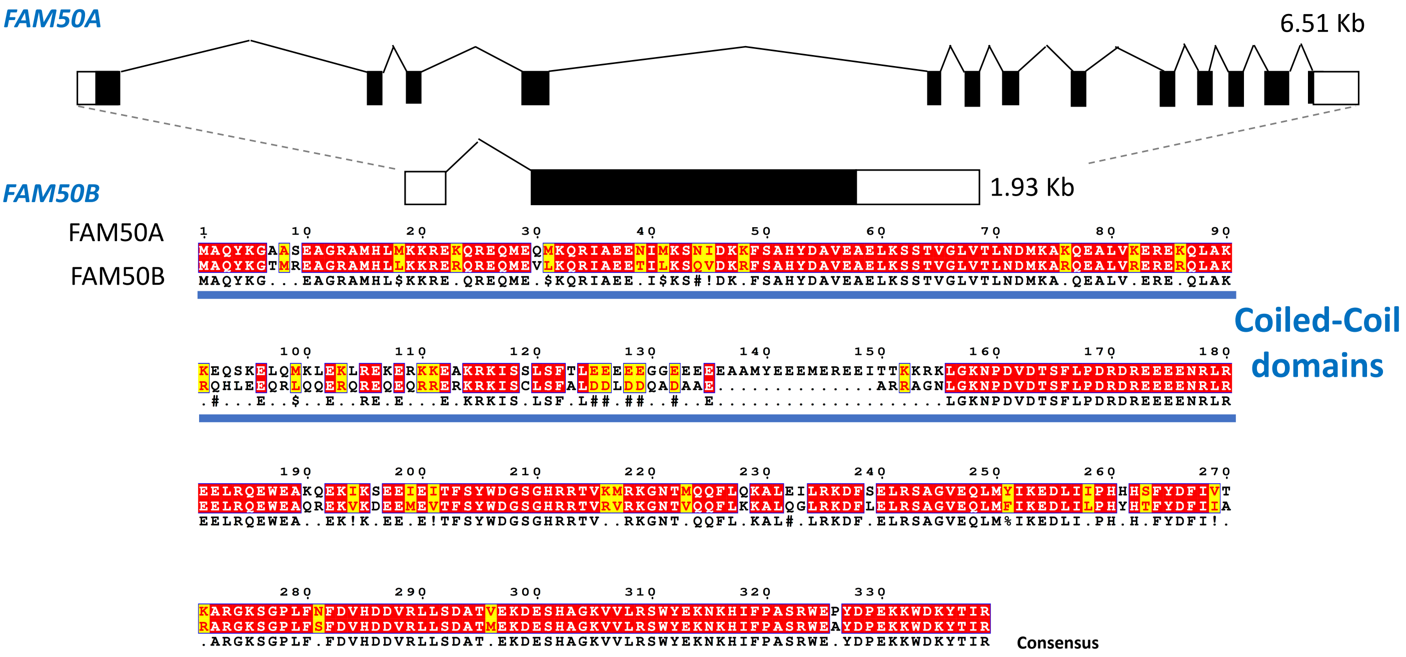

Supplementary Figure 13: Amino acid alignments of FAM50A and FAM50B. The coiled-coil domain is indicated with conserved residues highlighted in red. Above the alignment is a picture of the gene structures. The translations are of the transcripts: **ENST00000393600.8** & **ENST00000380272.3**.

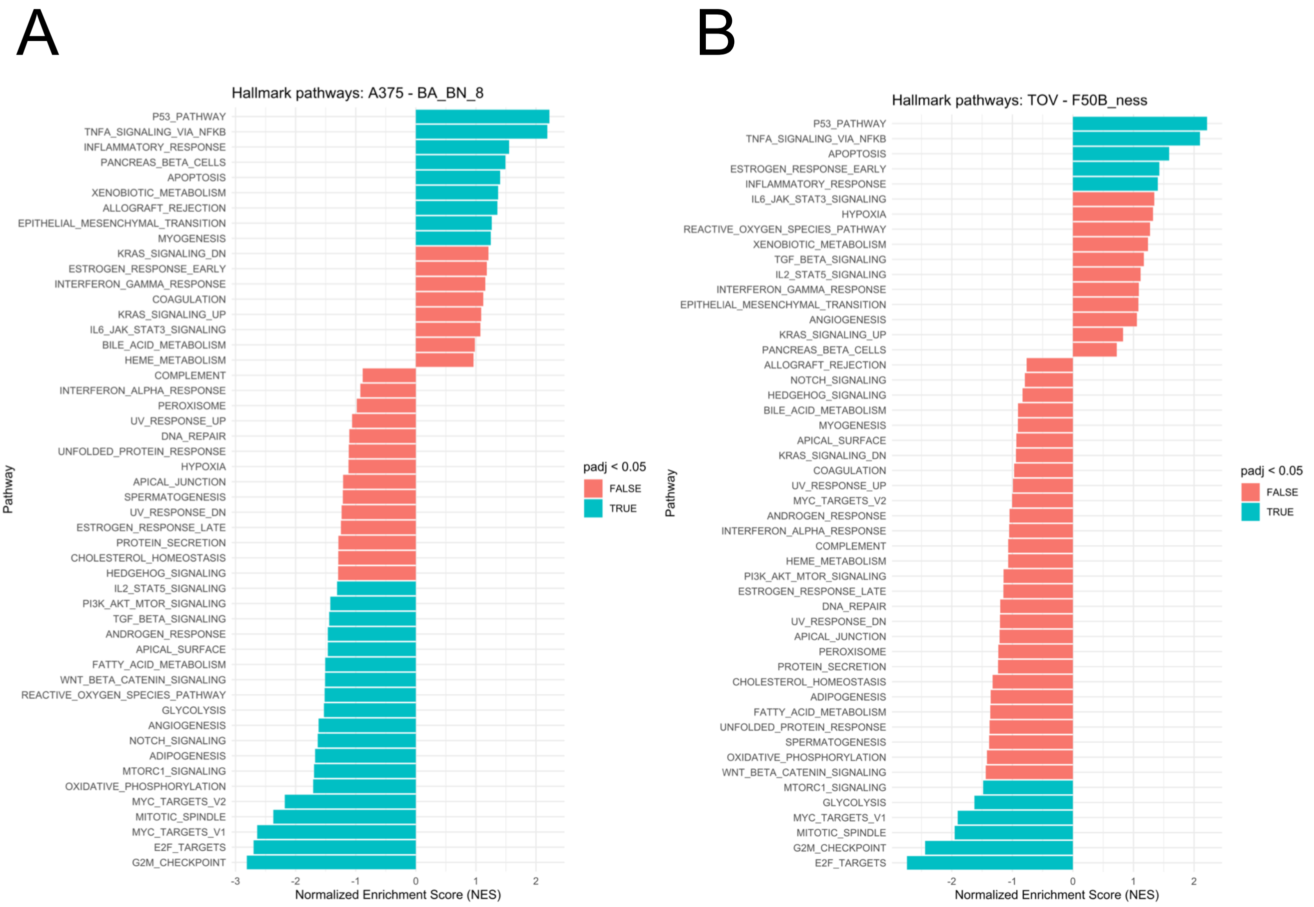

Supplementary Figure 14: Transcriptomic changes associated with *FAM50A/FAM50B* loss. (A). A375-*FAM50B* knockout cells (*F50B-*) were transduced with a *FAM50A* gRNA containing lentivirus or non-essential (*AIPL1*) gRNA containing lentivirus and cells collected 8 days after transduction. These cells constitutively express Cas9 as described in the **Methods**. (B). TOV21G cells, which do not express *FAM50B*, were transduced with a *FAM50A* gRNA containing lentivirus or non-essential (*AIPL1*) gRNA containing lentivirus and cells collected 8 days after transduction. These cells constitutively expressed Cas9 as described in the methods. Analysis was performed as described in the **Methods**. P values were adjusted for multiple testing with the Benjamini-Hochberg multiple test correction. These data are the result of three independent transductions.

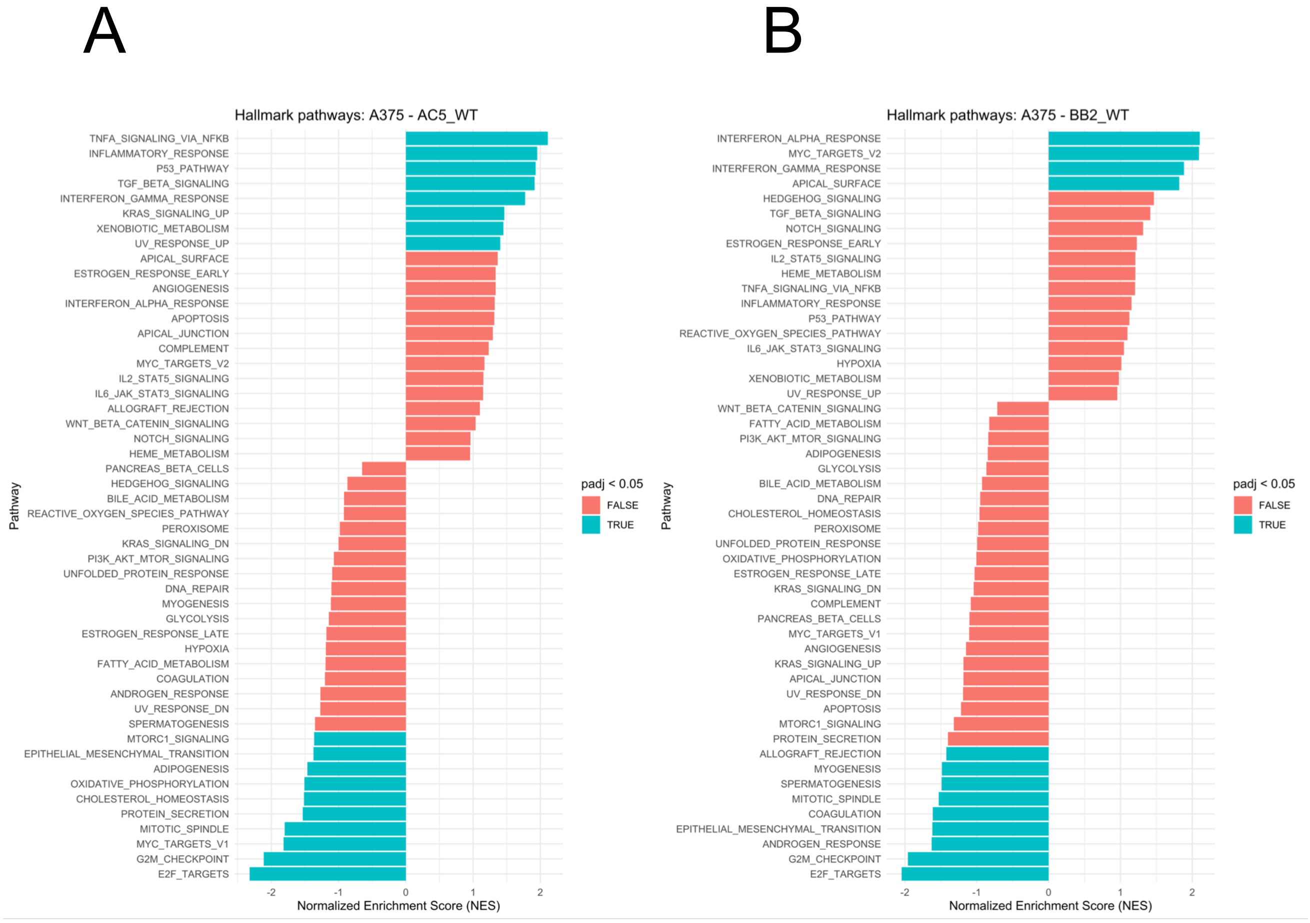

Supplementary Figure 15: Transcriptomic changes associated with loss of either *FAM50A* or *FAM50B* in A375 cells. (A). A375-*FAM50A* knockout (*F50A*-) or (B) A375-*FAM50B* knockout (*F50B*-) cells (stable knockout clones of *FAM50A* and *FAM50B*, respectively) were cultured for 7 days before RNA was collected for expression profiling in comparison to unmodified A375 cells. The figures show analysis of gene expression changes to define pathways that were statistically significantly altered. Analysis was performed as described in the **Methods**. P values were adjusted for multiple testing with the Benjamini-Hochberg multiple test correction. These data are the result of three independent transductions.
