# Supplementary material for: Combinatorial CRISPR screen identifies fitness effects of gene paralogues
Source: Nat Commun. 2021 Feb 26;12:1302. doi: 10.1038/s41467-021-21478-9 (PMC7910459; doi:10.1038/s41467-021-21478-9)
Supplement: Supplementary file 3 — Description of Additional Supplementary Files [file 41467_2021_21478_MOESM3_ESM.pdf]

Description of Additional Supplementary Files

Title: Supplementary Data 1

Description: Gene pairs used in the screen and analysis results.

Title: Supplementary Data 2

Description: Oligos used in this project.

Title: Supplementary Data 3

Description: Gene pairs and associated gene symbols.

Title: Supplementary Data 4

Description: gRNAs used for library construction.

Title: Supplementary Data 5

Description: Statistically significant gene pairs after filtering.

Title: Supplementary Data 6

Description: TCGA tumour expression levels for FAM50B.

Title: Supplementary Data 7

Description: Genetic interactions found in this study and their overlap with other studies.

Title: Supplementary Data 8

Description: FAM50A lethality scores and FAM50B gene expression across a panel of cancer cell lines.
