# Supplementary material for: Combinatorial CRISPR screen identifies fitness effects of gene paralogues
Source: Nat Commun. 2021 Feb 26;12:1302. doi: 10.1038/s41467-021-21478-9 (PMC7910459; doi:10.1038/s41467-021-21478-9)
Supplement: Supplementary file 12 — Reporting Summary [file 41467_2021_21478_MOESM12_ESM.pdf]

nature research

Corresponding author(s): David Adams: NCOMMS-20-13240

Last updated by author(s): Nov 14, 2020

Reporting Summary

Nature Research wishes to improve the reproducibility of the work that we publish. This form provides structure for consistency and transparency in reporting. For further information on Nature Research policies, see our Editorial Policies and the Editorial Policy Checklist.

Statistics

For all statistical analyses, confirm that the following items are present in the figure legend, table legend, main text, or Methods section.

n/a	Confirmed	
☐	☒	The exact sample size (*n*) for each experimental group/condition, given as a discrete number and unit of measurement
☐	☒	A statement on whether measurements were taken from distinct samples or whether the same sample was measured repeatedly
☐	☒	The statistical test(s) used AND whether they are one- or two-sided<br>*Only common tests should be described solely by name; describe more complex techniques in the Methods section.*
☐	☒	A description of all covariates tested
☐	☒	A description of any assumptions or corrections, such as tests of normality and adjustment for multiple comparisons
☐	☒	A full description of the statistical parameters including central tendency (e.g. means) or other basic estimates (e.g. regression coefficient) AND variation (e.g. standard deviation) or associated estimates of uncertainty (e.g. confidence intervals)
☐	☒	For null hypothesis testing, the test statistic (e.g. *F*, *t*, *r*) with confidence intervals, effect sizes, degrees of freedom and *P* value noted<br>*Give P values as exact values whenever suitable.*
☒	☐	For Bayesian analysis, information on the choice of priors and Markov chain Monte Carlo settings
☒	☐	For hierarchical and complex designs, identification of the appropriate level for tests and full reporting of outcomes
☒	☐	Estimates of effect sizes (e.g. Cohen's *d*, Pearson's *r*), indicating how they were calculated

Our web collection on statistics for biologists contains articles on many of the points above.

Software and code

Policy information about availability of computer code

Data collection	N/A
Data analysis	Kallisto quantifier (v 0.44.0)<br>DESeq2 (3.12)<br>Bioconductor version: Release (3.11) (fgsea)<br>GraphPad Version v7.04 & 8.4.3<br>R version 3.6.3<br>FlowJo v10.4.2<br>No software was used for data collection.

For manuscripts utilizing custom algorithms or software that are central to the research but not yet described in published literature, software must be made available to editors and reviewers. We strongly encourage code deposition in a community repository (e.g. GitHub). See the Nature Research guidelines for submitting code & software for further information.

Data

Policy information about availability of data

All manuscripts must include a data availability statement. This statement should provide the following information, where applicable:

- Accession codes, unique identifiers, or web links for publicly available datasets
- A list of figures that have associated raw data
- A description of any restrictions on data availability

The raw sequencing data are available for download from the European Genome-Phenome Archive under study accessions ERP108391, EGAS00001004156 and

EGAS00001002117. All other data can be found in the Supplementary Tables of this paper or in the Source Data file.

Field-specific reporting

Please select the one below that is the best fit for your research. If you are not sure, read the appropriate sections before making your selection.

- [x] Life sciences
- [ ] Behavioural & social sciences
- [ ] Ecological, evolutionary & environmental sciences

For a reference copy of the document with all sections, see nature.com/documents/nr-reporting-summary-flat.pdf

Life sciences study design

All studies must disclose on these points even when the disclosure is negative.

Sample size	No formal statistical method was used to predetermine sample sizes because we had no a priori information on the expected effect. For animal experiments we used the ARRIVE guidelines and a resource equation approach, estimating a 25% engraftment failure rate. https://www.ncbi.nlm.nih.gov/pmc/articles/PMC5772820/.
Data exclusions	No data was excluded. All datapoints are plotted.
Replication	All of the studies were biologically replicated at least three times on different days except for the initial screen which was performed in triplicate per cell line, which is customary for studies of this type, and the competitive growth assays in Figure 2C and Supp Figures 7&8, which were performed as three independent transductions of each cell line, with each of these experiments being performed on three different cell lines. All data points were plotted and not data was excluded. All replication attempts for all studies are shown in the manuscript. The surveyor assay to illustrate gRNA cutting (Supp Figure 7) and the Western blot to show A375-FAM50A knockout cells had lost FAM50A protein (Supp Figure 11) were performed once. Three cultures of both FAM50A and FAM50B knockout A375 cell lines were transcriptome sequenced and all cultures showed clear loss of either FAM50A or FAM50B transcript.
Randomization	For the animal studies shown in Figure 3 tumour cells were implanted into mice and the mice were randomized into two groups for treatment by random selection (drawing them from a hat).
Blinding	No blinding was performed except that the person measuring the tumours in Figure 4 was unaware of the treatment groups. Blinding was not performed for the other experiments because none of the studies were randomized control trials.

Reporting for specific materials, systems and methods

We require information from authors about some types of materials, experimental systems and methods used in many studies. Here, indicate whether each material, system or method listed is relevant to your study. If you are not sure if a list item applies to your research, read the appropriate section before selecting a response.

Materials & experimental systems

n/a	Involved in the study
☐	☒ Antibodies
☐	☒ Eukaryotic cell lines
☒	☐ Palaeontology and archaeology
☐	☒ Animals and other organisms
☒	☐ Human research participants
☒	☐ Clinical data
☒	☐ Dual use research of concern

Methods

n/a	Involved in the study
☒	☐ ChIP-seq
☐	☒ Flow cytometry
☒	☐ MRI-based neuroimaging

Antibodies

Antibodies used	FAM50A. ABCAM. Rabbit monoclonal (ab186410)[Batch EPR12816]. 1:3000 dilution. Blocking buffer-Milk Rabbit<br>https://www.abcam.com/fam50axap5-antibody-epr12816-ab186410.html?productwalltab=abreviews&productWallTab=Abreviews<br>CD15-APC: Miltenyi, 130-098-008. CD15-APC used at 1:5 dilution.<br>CD33-PE: Miltenyi, 130-098-896. CD33-PE used at 1:5 dilution.<br>Vinculin SAB4200080; Sigma 1:3000 dilution. Blocking buffer-Milk Mouse<br>Anti-rabbit #7047 Cell Signalling 1:5000 dilution. Blocking buffer-Milk/BSA<br>Anti-mouse #7076 Cell Signalling 1:5000 dilution. Blocking buffer-Milk/BSA
Validation	We generated knockout cell lines for FAM50A and they no longer expressed the protein.<br>We generated double knockout CD33/CD15 cells and showed loss of expression by FACS.

Other validation notes:
FAM50A - reacts with mouse/human/rat FAM50A. Validated by manufacturer for WB, IHC and ICC/IF. Manufacturer used range of cell lines as a positive control. As above we generated FAM50A knockout clones and the antibody no longer detected any protein, acting as a negative control, whilst the wild type cell line had a strong band for FAM50A at the appropriate molecular weight.

Vinculin: This antibody has featured in over 1077 peer reviewed papers and is suitable for western blot, reacting with human vinculin.

CD33-PE - This antibody reacts with the human CD33 epitope and is validated for flow cytometry. Miltenyi has performed extended validation on this antibody, looking at sensitivity (using epitope competition assays) and specificity, with and without fixation.
Reference: Bernstein, I. D. et al. (1987) Treatment of acute myeloid leukemia cells in vitro with a monoclonal antibody recognizing a myeloid differentiation antigen allows normal progenitor cells to be expressed. J. Clin. Invest. 79(4): 1153-1159

CD15-APC - This antibody reacts with the human CD15 epitope and is validated for flow cytometry.
Reference: Choi, K.-D. et al. (2011) Hematopoietic differentiation and production of mature myeloid cells from human pluripotent stem cells. Nat. Protoc. 6(3): 296-313

Eukaryotic cell lines

Policy information about cell lines

Cell line source(s)	All the cell lines listed below were from the American-Type Culture Collection.<br><br>293T<br>A375<br>Mewo<br>Molm-13<br>TOV-21G<br>hTERT RPE-1<br>RKO
Authentication	All cell lines were STR profiled and found to match perfectly to published STR profiles.
Mycoplasma contamination	All cell lines were mycoplasma tested and found to be contamination free.
Commonly misidentified lines (See ICLAC register)	No such cell lines were used.

Animals and other organisms

Policy information about studies involving animals; ARRIVE guidelines recommended for reporting animal research

Laboratory animals	Mice, NOD-SCID. Both males and females; 6-8 weeks old. Full details are provided in the figure legends. Mice were maintained in a specific pathogen-free unit on a 12h light:12h dark cycle with lights off at 19:30 and no twilight period. The ambient temperature was 21±2°C, and the humidity is 55±10%. Mice were housed using a stocking density of 3–5 mice per cage (overall dimensions of caging: 365×207×140mm3 (length×width×height), floor area 530cm2) in individually ventilated caging (Tecniplast, Sealsafe 1284L) receiving 60 air changes per hour. In addition to Aspen bedding substrate, standard environmental enrichment of two Nestlets, a cardboard fun tunnel, and three wooden chew blocks are provided. Mice were given water and diet ad libitum.
Wild animals	No wild animals were used.
Field-collected samples	No field collected samples were used.
Ethics oversight	Home Office Project license: P6B8058BO. Procedures were further approved by the Animal Welfare Ethical Review Body (AWERB) of the Welcome Trust Sanger Institute. Our animal studies are fully compliant with the ARRIVE guidelines.

Note that full information on the approval of the study protocol must also be provided in the manuscript.

Flow Cytometry

Plots

Confirm that:

- [x] The axis labels state the marker and fluorochrome used (e.g. CD4-FITC).
- [x] The axis scales are clearly visible. Include numbers along axes only for bottom left plot of group (a 'group' is an analysis of identical markers).
- [x] All plots are contour plots with outliers or pseudocolor plots.
- [x] A numerical value for number of cells or percentage (with statistics) is provided.

Methodology

Sample preparation	For the CD15/CD33 experiment:At a given time point, 1 million cells were pelleted (500g, 3 minutes) before washing twice with 1ml FACS buffer. Cells were subsequently re-suspended in 30µl FACS buffer/20µl FcR blocking reagent and incubated at 4oC for 5 minutes. 30µl FACS buffer/10µl CD33- PE /10µl CD15-APC was added and cells were incubated at 4oC, protected from light for 20 minutes. 1ml FACS buffer was subsequently added to the cells before pelleting, discarding the supernatant and resuspending the cells in 500µl FACS buffer contain- ing DAPI at a final concentration of 0.2µg/ml. Fluorescence was read on the LSRII Flow cyometer (BD) reading PE (488 (575/26)); APC (633 (660/20)) and DAPI (355 (450/50)). PBS, 0.5% FBS, 2mM EDTA, 0.1% sodium azide.<br>For the validation experiments: cells were trypsinised and re-suspended in standard medium. They were then centrifuged (500g/3 minutes) and the pellet fully re-suspended in 4% Paraformaldehye/PBS (Sigma, F8775) in order to fix the cells. The samples were then incubated for 15 minutes at room temperature. They were then centrifuged again (500g, 3 minutes), re-suspended 500µl of PBS/1% BSA (Sigma, A2153)<br>For the apoptosis experiment: cells were harvested alongside the media they were growing in. Cells were then pelleted (500g; 3mins) and the supernatant aspirated. Cells were re-suspended in 300µl media/1µl CaspGLOW and incubated for 1 hour at 37oC/5% CO2. 3mls of PBS was then added and cells were pelleted (500g; 3mins) before resuspending in 100µl live/dead e780 viability indicator (Invitrogen 65-0865), diluted to 1:2000 in PBS. Cells were incubated at 5oC at room temperature before 3mls PBS was added. Cells were then pelleted and re-suspended in 500µl of FACS buffer.
Instrument	LSRII Flow cyometer (BD)
Software	FlowJo. Version 10.
Cell population abundance	We didn't use flow sorting.
Gating strategy	Cells were identified using SSC(A) and FSC(A). Single cells were then gated using FSC(A) and FSC(W). All single cells gated were used in the analysis.

☒ Tick this box to confirm that a figure exemplifying the gating strategy is provided in the Supplementary Information.
